# Assessing Exposure to Organophosphorus Pesticides by Biomonitoring in Epidemiologic Studies of Birth Outcomes

**DOI:** 10.1289/ehp.7490

**Published:** 2005-01-10

**Authors:** Larry L. Needham

**Affiliations:** Centers for Disease Control and Prevention, Atlanta, Georgia, USA

**Keywords:** biomonitoring, chlorpyrifos, cord blood, organophosphorus insecticides, urine

## Abstract

For epidemiologic studies that evaluate the relation between potential exposures to environmental chemicals and adverse outcomes, accurate assessments of exposures and health outcomes are needed. Three prospective cohort studies recently evaluated the relation between exposure, as assessed by biomonitoring, of pregnant women to organophosphorus pesticides and several birth outcomes. Here these three studies are compared in terms of the exposure scenarios and exposure assessments. The primary focus is on the exposure assessments, all of which employ biomonitoring but use different approaches, which may contribute to the different findings. These approaches and how they may contribute to different relations between exposure and birth outcomes are examined.

Three recent studies (Berkowitz et al. 2003, 2004; Eskenazi et al. 2004; Perera et al. 2003; Whyatt et al. 2004) appearing in *Environmental Health Perspectives* examined the relation between pregnant women’s exposures to selected pesticides and birth outcomes. The participants in each of these studies were likely exposed to many classes of pesticides as well as to other environmental chemicals, but the primary exposure focus of these studies was on organophosphorus pesticides. Of the approximately 40 organophosphorus pesticides registered by the U.S. Environmental Protection Agency (EPA), chlorpyrifos was used in large amounts in all three exposure scenarios, and among the pesticides measured in these studies, chlorpyrifos or its metabolite 3,5,6-trichloro-2-pyridinol (TCPy) was detected in the highest concentrations in the biologic specimens tested. Exposure to chlorpyrifos was assessed by measuring it in umbilical cord and maternal blood samples (Whyatt et al. 2004) and by measuring its specific metabolite, TCPy, in urine (Berkowitz et al. 2004; Eskenazi et al. 2004), and is included in the measurements of a suite of nonspecific metabolites [dialkyl phosphates (DAPs)] of organophosphorus pesticides (Eskenazi et al. 2004). All three studies statistically examined the relation between pesticide exposures and several birth outcomes, although, like the exposure assessment methods, the birth outcomes examined were not identical across these studies. The significant relations reported between their exposure assessments and birth outcomes included decreased birth size (Whyatt et al. 2004), decreased head circumference with levels of paraoxonase 1 (PON1) activity as a modifier (Berkowitz et al. 2004), and decreased gestational age at birth (Eskenazi et al. 2004). Further details on the exposure scenarios, exposure assessments in relation to birth outcomes, and the data analyses are examined.

## Exposure Settings

The pathways for the organophosphorus insecticide exposures differ between the California (Eskenazi et al. 2004) and New York studies (Berkowitz et al. 2004; Whyatt et al. 2004) but are probably similar for the two New York City studies. The major sources of organophosphorus insecticides throughout pregnancy in the New York studies were assumed to be recurrent indoor broadcast sprayings, primarily of chlorpyrifos (Landrigan et al. 1999). The fate of chlorpyrifos in a setting similar to that of the New York City studies was reported by Gurunathan et al. (1998). They found that after spraying chlorpyrifos, air concentrations peaked in a two-phase process: an aerosolized particle phase with chlorpyrifos concentration peaking on surface areas after 36 hr and a gas phase that began 12 hr after application and continued for at least 2 weeks, with chlorpyrifos concentrations peaking on surface areas at 72 hr and then declining. Of course, the degree of ventilation in a particular residence would affect the amount of chlorpyrifos in the air. Whyatt et al. (2004) indicated that the insecticides were sprayed repeatedly in the residences of the pregnant women in their New York study. Therefore, the chances for repeated exposures were high.

In the California study (Eskenazi et al. 2004), the primary exposure focus was agricultural applications in the Salinas Valley of several pesticides, pesticide drift from the spraying, and the “bringing home” of the pesticides on workers’ clothing; however, pesticides were also potentially used indoors, for landscape maintenance, for insect control, and for prevention of structural damage (Castorina et al. 2003). Eskenazi et al. (2004) noted that during pregnancy 28% of the California women worked in the fields, another 14% worked at other agricultural jobs, and a total of 85% had agricultural workers living in their homes.

**Figure 1:**
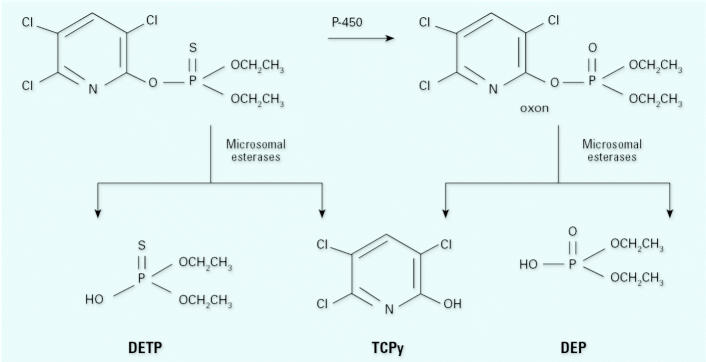
Human metabolism of chlorpyrifos.

Before proceeding further, it is important to understand the human metabolism and environmental degradation of organophosphorus insecticides, by using chlorpyrifos as an example (Figure 1). Chlorpyrifos is bioactivated by cytochrome P450–dependent desulfuration in the liver to chlorpyrifosoxon. This oxon is rapidly hydrolyzed to its specific metabolite TCPy and to diethylphosphate (DEP) by microsomal esterases, which include PON1 and chlorpyrifos oxonase, or by nonenzymatic hydrolysis (Racke 1993). Alternatively, chlorpyrifos is dearylated to form TCPy and diethylthiophosphate (DETP) by microsomal enzymes or by nonenzymatic hydrolysis. A complicating factor in interpreting chlorpyrifos metabolite concentrations in urine is that these human metabolites and their environmental degradates are the same chemicals. Hence, assuming these degradates are excreted unchanged, measuring chlorpyrifos metabolites in urine does not distinguish exposure to chlorpyrifos from exposure to the environmental degradates. In general, organophosphorus pesticides are metabolized by these routes, and about 75% of the 40 or so organophosphorus pesticides that are registered by the U.S. EPA are biotransformed to DETP, DEP, or four other DAPs that are excreted in urine and measured in the same analytical method (Bravo et al. 2004). Castorina et al. (2003) noted that although many organophosphorus pesticides were used in the Salinas Valley, the applied amount of chlorpyrifos was similar to that of oxydemeton-methyl but less than that of either malathion or diazinon for each of the years 1999–2001. Eskenazi et al. (2004) examined urinary levels of the specific metabolites of these pesticides, except for oxydemeton-methyl, and found TCPy, the chlorpyrifos metabolite, much more frequently and in much higher concentrations in their cohort than the specific metabolites of malathion (malathion dicarboxylic acid; MDA) and diazinon (2-isopropyl-4-methyl-6-hydroxy-pyrimidine); for example, the median urinary concentrations and the frequency of results above the method’s limit of detection (LOD) for TCPy and MDA were 3.3 μg/L and 77% and 0.2 μg/L and 30%, respectively. Acephate was also used in the Salinas Valley, but exposure to it was not examined

Thus, the organophosphorus insecticides applied and the environmental settings in New York and California differed. From the “exposure scenario” information, one could assume that the amount and rate of exposure to pesticides and the type of pesticides were similar in the two New York studies, but direct comparisons of exposure with the California study are difficult. Compounding this difficulty, within the California study population, Eskenazi et al. (2004) noted several exposure scenarios. Some general comparisons can be made, however, between indoor and outdoor settings. Because of the environmental conditions, such as availability of water and ultraviolet light, which promote hydrolysis, organophosphorus pesticides tend to have a longer half-life indoors compared with outdoors. Therefore, in indoor settings, the ratio of the concentrations of parent pesticide to the less biologically active environmental degradates of the pesticide was likely higher than in outdoor settings. Also, a higher percentage of the cumulative exposures to organophosphorus pesticides in the indoor settings was probably derived from inhalation because of the pesticides’ longer duration in the homes and the amount of time people spend indoors, including sleeping; thus, the New York City populations were probably more continuously exposed to the applied pesticides. In addition, the indoor broadcast spraying may have led to increased nondietary ingestion of chlorpyrifos by New York participants compared with the participants in the California study. In California, the bulk of the pesticides were applied in agricultural fields, although some indoor use for insect control occurred. Therefore, especially among the California agricultural workers, more dermal exposure may have been experienced.

## Exposure Assessment

**Figure 2:**
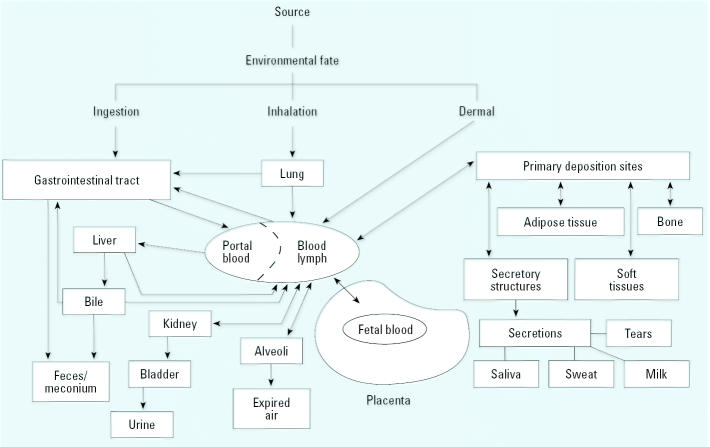
Transfer of environmental chemicals from pregnant woman to fetus.

The overarching issue regarding the exposure assessment concerns the dose of the biologically active chemical at the target site, which in these studies is the developing fetus. Figure 2 shows the three routes of exposure for and the toxicokinetics (absorption, distribution, metabolism, and elimination) of a nonpersistent pesticide in the pregnant woman. The route of exposure can play a role in the relative amount of fetal exposure to chlorpyrifos and other organophosphorus insecticides. For example, if the route is ingestion, much of the chlorpyrifos may be metabolized to TCPy in the first pass effect before chlorpyrifos reaches the maternal systemic blood supply and hence the placenta (Figure 2). After oral administration of chlorpyrifos to the rat, Timchalk et al. (2002) indicated that chlorpyrifos was readily absorbed and metabolized, with peak blood chlorpyrifos levels observed by 3 hr after dosing. The amount of unmetabolized chlorpyrifos in blood after dosing was low: 100-fold lower than TCPy, according to rat and human studies of Nolan et al. (1984) and Timchalk et al. (2002). In addition, Nolan et al. (1984) found that the terminal elimination half-life of TCPy in human blood was 27 hr and that 70% of the oral dose of chlorpyrifos was eliminated as TCPy in urine within 4 days of exposure. Timchalk et al. (2002) could not detect the chlorpyrifosoxon after the oral administration of chlorpyrifos at 0.5–2 mg/kg in 12 human volunteers. However, regardless of the exposure route, the absorbed dose is mostly metabolized, and the TCPy metabolite is distributed throughout the body in the blood and eliminated through the kidney (Figure 2). Because the toxicokinetics of administered TCPy or the DAPs metabolites have not been studied in similar detail as chlorpyrifos, we do not know their toxicokinetics, particularly if their elimination products are TCPy or DAPs or some other chemicals. For example, findings from one animal study suggest that exposure to DEP would result predominantly in the excretion of inorganic phosphate (Imaizumi et al. 1993), but the metabolic fate of DAPs is not known for humans. If TCPy and DAPs are the primary excreted chemicals after exposure to them, measurement of these metabolites may overestimate exposure to chlorpyrifos or other organophosphorus insecticides depending on the exposure pathway (Morgan et al. 2004).

As noted in Figure 2, chlorpyrifos is distributed within the body by the systemic circulatory system. The amount of parent chlorpyrifos in the blood of the women in these three studies obviously depended on the concentration of chlorpyrifos and the route in the most recent exposure, the exposure rate, the time interval since the last exposure, and their individual characteristics, including pregnancy, which affect the toxicokinetics of chlorpyrifos. However, the amount of parent chlorpyrifos in maternal blood also depended on the amount of chlorpyrifos equilibrated, as a result of previous exposures, with its concentration in adipose tissue, which is a storage site for chlorpyrifos and other lipophilic chemicals. Because of its lipophilicity (log *K*_ow_ = 4.82) (McCall et al. 1980), chlorpyrifos tends to store in the body’s adipose tissue, but because of its high rate of metabolism, chlorpyrifos has a half-life in the adipose tissue of rats of only 62 hr (Smith et al. 1967). In humans, concentrations of chlorpyrifos in maternal blood and adipose tissue are likely to be in steady state with each other and with its concentration in fetal blood unless the blood samples are taken very soon after exposure to chlorpyrifos. Whyatt et al. (2004) showed that chlorpyrifos concentrations in maternal and cord plasma samples were correlated (*r* = 0.76; *p* < 0.001, Spearman’s rank). This finding suggests the potential use of maternal blood for assessing exposure to organophosphorus insecticides, although Whyatt et al. (2004) did not describe whether a relation existed between maternal blood concentrations of chlorpyrifos and these birth outcomes. The major advantages of using maternal blood rather than cord blood are ease of collection and ability to obtain multiple samples to evaluate concentrations at different times during gestation. Of course, the main advantage of using cord blood rather than maternal blood is that the former is in direct contact with the fetus, the target site in these studies. Therefore, for assessing effects to the fetus, maternal or cord blood concentrations of chlorpyrifos may be better indicators than the less direct indicators of maternal urine TCPy concentrations. The reasons for this include the use of the blood samples as surrogates for the fetus and the specificity of measuring the parent chemical (Lu et al. 2004; Morgan et al. 2004; Wilson et al. 2003). That Whyatt et al. (2004) showed the relation for cord blood chlorpyrifos levels with newborn growth decrements, but were unable to associate them with self-reported use or air monitoring measurements, suggests that measures more proximate to the site of action may be more predictive of an effect, a main tenet of biomonitoring.

Measuring urine levels of nonpersistent chemicals and their metabolites is widely accepted in occupational and environmental settings, so this approach warrants consideration. In general, the measurement of metabolites of organophosphorus metabolites in urine allows several advantages: assessing an integrated exposure when blood concentrations are changing, allowing a more detectable measure of exposure assessment because of the higher urinary concentrations, allowing multiple determinations throughout pregnancy with noninvasive sampling, in some cases measuring an active metabolite, assessing exposure to one specific organophosphorus pesticide or perhaps to a few (e.g., measurement of TCPy potentially assesses exposure to both chlorpyrifos and chlorpyrifos-methyl), and assessing exposure to multiple organophosphorus insecticides [about three-fourths of the U.S. EPA–registered organophosphorus pesticides form six DAP metabolites (Barr et al. 1999; Barr and Needham 2002; Needham and Sexton 2000)]. However, measurement of TCPy in urine, as was done in the studies by Eskenazi et al. (2004) and Berkowitz et al. (2004), does not differentiate among exposure to TCPy, chlorpyrifos, and chlorpyrifos-methyl.

Another advantage of analyzing urine for metabolites of chlorpyrifos and other organo-phosphorus pesticides is that we are able to compare these concentrations with concentrations reported in the U.S. general population from the National Health and Nutrition Examination Survey (NHANES) [Centers for Disease Control and Prevention (CDC) 2003; Barr et al. 2004; Needham et al., in press]. However, these NHANES data contain only 96 pregnant women, and we do not know the effect of pregnancy on the toxicokinetics (rates of absorption, distribution, metabolism, and elimination) of chlorpyrifos, so urinary TCPy concentrations in pregnant women and the concentrations in the NHANES data should be compared with caution. These U.S. data show that most of the U.S. general population is exposed to chlorpyrifos, chlorpyrifos-methyl, their oxons, TCPy, or a combination of these chemicals. The median and geometric mean urinary TCPy concentrations in 1,022 samples from females 6–59 years of age in 1999–2000 were 1.50 and 1.63 μg/L, respectively. TCPy was detected in > 91% of the NHANES samples; this high percentage of detectable concentrations implies ubiquitous exposure, probably primarily through the food chain. In these three research studies, however, exposure from other environmental media and routes might predominate. In the studies by Berkowitz et al. (2004) and Eskenazi et al. (2004), exposure assessment methods included the measurement of urinary concentrations of TCPy at one and two times during pregnancy, respectively. The median levels of urinary TCPy differed between the two studies: 7.6 μg/L for the Berkowitz et al. study (2004) and 3.3 μg/L for the Eskenazi et al. study (2004). Because of the large number (57%) of concentrations below the method’s LOD and the imputation of concentrations below the LOD, the median value of 7.6 μg/L was below the reported LOD of 11.0 μg/L for the analytical method (high-performance liquid chromatography–ultraviolet light detection) that Berkowitz et al. (2004) employed. Such imputations are subject to a great deal of inaccuracy, and perhaps this is one reason Berkowitz et al. (2004) compared newborn outcome data and exposure data by dividing the urinary TCPy concentrations into two groups: mothers with urinary TCPy concentrations below the LOD of 11.0 μg/L (57%) and those with concentrations equal to or above the LOD (43%). Of course, this value of 11.0 μg/L was chosen on an analytical, rather than a biologic, basis and thus may have no biologic relevance. Compared with the TCPy concentrations found in the NHANES data, the concentration of 11.0 μg/L is above the 95th percentile of all females 6–59 years of age (95th percentile = 10.0 μg/L), above the 95th percentile for all Mexican Americans (7.40 μg/L), above the 95th percentile for all non-Hispanic whites (10 μg/L), but below the 95th percentile for all non-Hispanic blacks (13.0 μg/L) (CDC 2003). In the study by Berkowitz et al. (2004), those mothers with TCPy urinary concentrations above 11.0 μg/L and with low PON1 activity tended to give birth to infants with smaller head circumference.

Eskenazi et al. (2004) compared the newborn outcome data with maternal TCPy concentrations below their LOD (0.26 μg/L), with TCPy concentrations above the detection limit but below the median urinary TCPy concentration of 3.3 μg/L, and with TCPy concentrations equal to or greater than that median concentration. As in the Berkowitz et al. (2004) study, no known biologic basis exists for using this 3.3 μg/L value, although the median value is often used for such comparisons. The median urinary levels of TCPy in the Eskenazi et al. study (2004) are similar to the 75th percentile in the U.S. population, in which the 75th percentile for all females was 3.30 μg/L and for all Mexican Americans was 3.20 μg/L (CDC 2003). However, the percentage of TCPy urinary concentrations above the LOD in the NHANES population was higher (91%) than in the Eskenazi et al. study (76%) even though Eskenazi et al. (2004) reported using a slightly lower LOD (0.26 vs. 0.4 μg/L); the Eskenazi et al. (2004) and the NHANES urinary metabolite data were measured by high-performance liquid chromatography–tandem mass spectrometry in the CDC laboratory.

As mentioned above, the California setting involved agricultural applications of several organophosphorus insecticides; therefore, in addition to measuring more specific metabolites, such as TCPy, Eskenazi et al. (2004) measured the nonspecific DAPs, which are metabolites of most of the organophosphorus pesticides but not of all (e.g., acephate). The median levels of the dimethyl and diethyl phosphates for adults, females, and Mexican Americans are slightly less than the 75th percentiles of the U.S. NHANES estimates (CDC 2003). For example, Eskenazi et al. (2004) in their study in the Salinas Valley reported the median concentrations of the sum of the three dimethyl phosphates was 101 nmol/L. In comparison, for the 75th percentile for all Mexican Americans in NHANES, the concentrations were as follows: dimethylphosphate, 30.2; dimethylthiophosphate, 70.4; and dimethyldithiophosphate, 11.4 nmol/L. Similarly, for the diethyl phosphates, Eskenazi et al. (2004) reported a median concentration of 22 nmol/L. In NHANES, the concentrations for the 75th percentile for all Mexican Americans were as follows: DEP, 26.6; DETP, 4.9; and diethyldithiophosphate, 1.7 nmol/L. It would be advantageous to have comparison data for pregnant women and for the relevant age groups from NHANES, but nonetheless these DAPs and TCPy data are consistent in indicating that the metabolite data for the California population were approximately in the 75th percentile of various NHANES populations.

The studies by Perera et al. (2003) and Whyatt et al. (2004) also used multiple regression analyses to evaluate their blood chlorpyrifos data. If the data indicated an association between concentrations of chlorpyrifos and a newborn outcome, fetal chlorpyrifos concentrations were categorized into four exposure groups to evaluate the dose–response relations. In so doing, the researchers observed the birth effects of shorter length and lower weight principally when comparing effects in newborns with concentrations of fetal blood chlorpyrifos below their LOD (31%) with the highest fetal blood chlorpyrifos concentrations.

## Conclusions

The intent of the three studies was to compare fetal exposures to selected insecticides, primarily organophosphorus insecticides, with various birth outcomes. The purpose of this commentary is to examine these studies and discuss reasons why they did not show consistent relationships between exposure and birth outcomes. Most likely, the different conclusions were not related to errors in birth outcome measurements, although one cannot assume that the birth outcome measurements are accurate because they were not standardized or quality controlled for these studies. Differences in toxicokinetics and toxicodynamics (i.e., essentially how people “handle” and “respond” to the chemical exposures) may play a role in the birth outcomes (Needham et al. 2004). Race/ethnicity can influence both a chemical’s toxicokinetics and toxicodynamics. Whyatt et al.’s (2004) study population self-identified as being African American or Dominican; Berkowitz et al.’s (2004) study population was categorized in increasing numbers as white, African American, and Hispanic; and Eskenazi et al.’s (2004) study population was primarily Mexican American. Berkowitz et al. (2004) reported that when maternal PON1 activity was taken into account, low maternal PON1 activity and TCPy levels above the LOD were associated with decreased head circumference; however, the effect of PON activity on the outcome measures was not evaluated in the other studies. It would be of interest to know whether low PON1 activity was dependent on race/ethnicity. Sex can also be a factor in influencing both toxicokinetics and toxicodynamics. Although the presence of different sexes is not a factor here, female rats have shown increased risk to chlorpyrifos-induced toxicity compared with male rats (Chambers and Chambers 1989; Sultatos 1991), but bulls have shown more toxic effects than steers (castrated bulls) (Haas et al. 1983).

Of course, the exposure setting can play a role. Although the primary pathways and routes of exposure differ in the New York and California studies, it is not possible to compare the relative degrees of exposure in the Whyatt et al. study (2004) with the other two studies because the exposure dosimeters differ so dramatically. In the California study, it appears that exposure to chlorpyrifos is less than in the New York Berkowitz et al. (2004) study, but both populations, especially the California population, were potentially exposed to many other organophosphorus pesticides that could have affected birth outcomes. Therefore, because of the different exposure assessments used and the inherent difficulties associated with assessing exposures to nonpersistent chemicals, it is not possible to reliably compare the degree of exposure across these studies.

As with any epidemiologic study, we must take advantage of the strengths and weaknesses of these three studies and design and conduct future studies that shed more light on the relation between maternal chlorpyrifos exposure and birth outcomes. The epidemiologic studies should be designed to ensure participation of a sufficient number of participants with a wide range of chlorpyrifos exposures. Multiple exposure assessments, including environmental measurements, should be conducted to allow comparison of these studies with the results of future studies and also to more fully assess *a*) fetal exposures using umbilical cord blood measurements of chlorpyrifos and TCPy, *b*) maternal blood concentrations of the same chemicals obtained during the second and third trimesters, and *c*) TCPy and DAP concentrations on volume and creatinine-adjusted bases in urine collected during the second and third trimesters and analyzed with PON1 activity. These biologic specimens should be taken in a uniform, controlled manner using a strict protocol. The analytical methods used should be compared to ensure that resulting data are of similar quality; thus, the laboratories involved should compare data and methods in terms of precision, accuracy, and LODs before the analyses of study samples. Also, the data should be analyzed using similar statistical approaches. Because the blood chlorpyrifos concentration depends somewhat on the equilibrium between its concentrations in adipose tissue and blood, the blood (maternal and cord) concentrations should be evaluated on both a concentration basis and a lipid basis, as is done when measuring serum dioxin levels; the lipid-based measurements would adjust the concentrations in the plasma according to the lipid content of the plasma. The lipid concentration of the plasma affects the equilibrium of chlorpyrifos between adipose tissue and plasma and thus the amount of potentially bioavailable chlorpyrifos. Factors such as pregnancy, recent ingestion of a meal, and endogenous factors can affect blood lipid concentrations (Phillips et al. 1989). To glean as much information as possible, researchers should collect maternal blood and first-morning void urine samples on the day after chlorpyrifos application as well as on a day well removed from the day of application. A problem in duplicating the exposure scenario of the New York studies is that the indoor application of chlorpyrifos is no longer lawful in the United States. An international site that allows duplication of the conditions that existed when the studies in New York were conducted may be needed for future human studies or, if possible, relevant stored samples from these three studies should be used.
